# Adipose triglyceride lipase–mediated lipid catabolism is essential for bronchiolar regeneration

**DOI:** 10.1172/jci.insight.149438

**Published:** 2022-05-09

**Authors:** Manu Manjunath Kanti, Isabelle Striessnig-Bina, Beatrix Irene Wieser, Silvia Schauer, Gerd Leitinger, Thomas O. Eichmann, Martina Schweiger, Margit Winkler, Elke Winter, Andrea Lana, Iris Kufferath, Leigh Matthew Marsh, Grazyna Kwapiszewska, Rudolf Zechner, Gerald Hoefler, Paul Willibald Vesely

**Affiliations:** 1 Diagnostic and Research Institute of Pathology, Medical University of Graz, Graz, Austria.; 2 BioTechMed-Graz, Graz, Austria.; 3 Division of Cell Biology, Histology, and Embryology, Gottfried Schatz Research Center, Medical University of Graz, Graz, Austria.; 4 Institute of Molecular Biosciences, University of Graz, Graz, Austria.; 5 Core Facility Mass Spectrometry, Medical University of Graz, Graz, Austria.; 6 Institute of Molecular Biotechnology, NAWI Graz, Graz University of Technology, Graz, Austria.; 7 Ludwig Boltzmann Institute for Lung Vascular Research, Graz, Austria.; 8 Otto Loewi Research Center, Medical University of Graz, Graz, Austria.; 9 Institute for Lung Health, Giessen, Germany.

**Keywords:** Metabolism, Pulmonology, Fatty acid oxidation, Mitochondria

## Abstract

The lung airways are constantly exposed to inhaled toxic substances, resulting in cellular damage that is repaired by local expansion of resident bronchiolar epithelial club cells. Disturbed bronchiolar epithelial damage repair lies at the core of many prevalent lung diseases, including chronic obstructive pulmonary disease, asthma, pulmonary fibrosis, and lung cancer. However, it is still not known how bronchiolar club cell energy metabolism contributes to this process. Here, we show that adipose triglyceride lipase (ATGL), the rate-limiting enzyme for intracellular lipolysis, is critical for normal club cell function in mice. Deletion of the gene encoding ATGL, *Pnpla2* (also known as *Atgl*), induced substantial triglyceride accumulation, decreased mitochondrial numbers, and decreased mitochondrial respiration in club cells. This defect manifested as bronchiolar epithelial thickening and increased airway resistance under baseline conditions. After naphthalene‑induced epithelial denudation, a regenerative defect was apparent. Mechanistically, dysfunctional PPARα lipid-signaling underlies this phenotype because (a) ATGL was needed for PPARα lipid-signaling in regenerating bronchioles and (b) administration of the specific PPARα agonist WY14643 restored normal bronchiolar club cell ultrastructure and regenerative potential. Our data emphasize the importance of the cellular energy metabolism for lung epithelial regeneration and highlight the significance of ATGL-mediated lipid catabolism for lung health.

## Graphical abstract

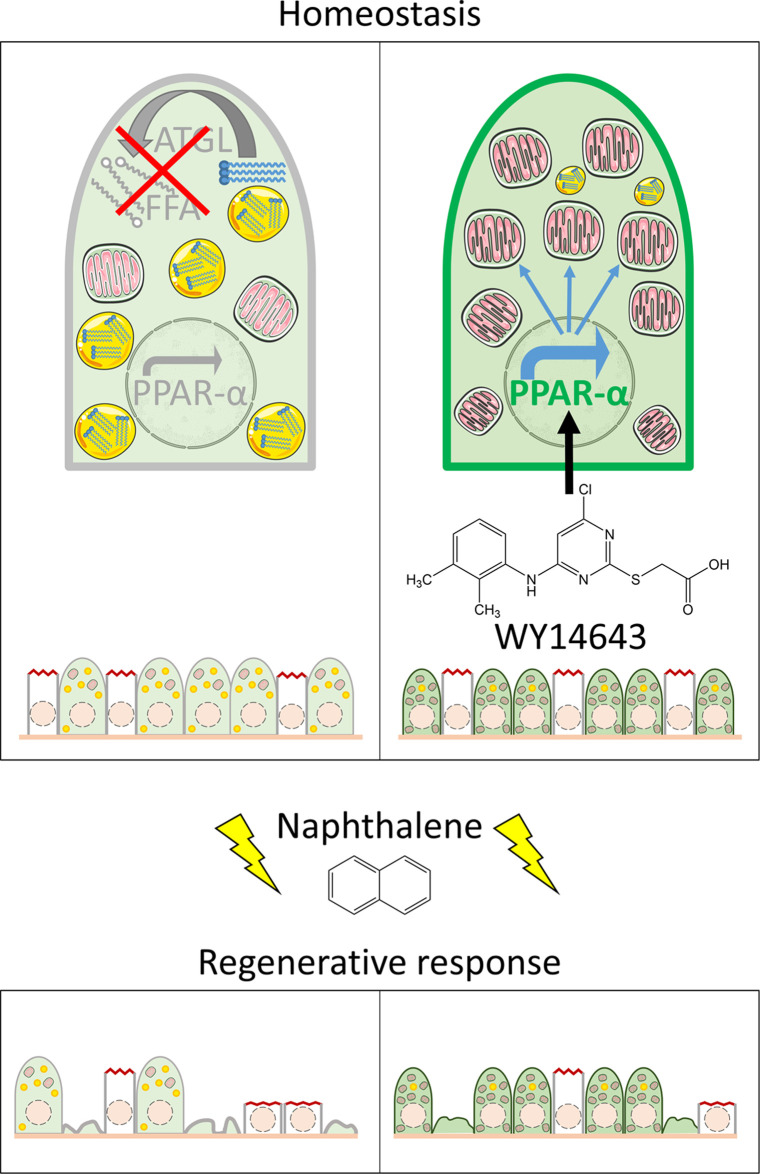

## Introduction

The lung airways are a main entry portal in many organisms. Bronchiolar epithelial cells are directly exposed to environmental toxins and insults from inhaled particulate matter that constantly cause epithelial injury. Bronchiolar epithelial repair is of critical importance for lung health because persistent injury is a significant factor for some of the most common lung diseases, including chronic obstructive pulmonary disease, asthma, pulmonary fibrosis, and lung cancer (1–5). As a consequence, the airways possess powerful detoxification and regeneration systems. In the upper tracheobronchial region, the airway epithelium is regenerated from basal cells that function as stem cells (6). In contrast, the lower airways of mice (<300 μm in diameter) are devoid of basal cells; here, the bronchiolar epithelium mainly consists of club cells and ciliated cells. Club cells are characterized by cytochrome P450 2F2 (CYP2F2) expression, and ciliated cells specifically have cilia positive for acetylated α-Tubulin (ac-αTUB). Club cells perform 3 major functions in the small airways of many higher vertebrates: (a) they are the main producers of secretory proteins necessary for the protective extracellular lining, (b) they metabolize environmental xenobiotics, and (c) club cells regenerate the bronchiolar epithelium after severe injury (7–9). Treatment of mice with the polycyclic aromatic hydrocarbon naphthalene (NA), for example, denudes the small airways by depleting club cells. NA is found in cigarette smoke and polluted air and is metabolically degraded through the CYP2F2 pathway in club cells. As a result, toxic epoxide intermediate metabolites accumulate and CYP2F2-expressing club cells perish within a day. At the same time, the bronchiolar basement membrane is protected through the nonproliferative spreading of ciliated cells. Within a few days, the small airway epithelium is then repopulated by the proliferative spreading of the remaining club cells (7, 10–12).

The ability to deal with such diverse tasks demands high metabolic plasticity from club cells. Regulation of the cellular energy metabolism critically involves fine tuning of nutrient uptake, energy conversion, energy storage, and stored energy retrieval (13–15). Eukaryotic cells efficiently store energy in the form of triglyceride in cellular lipid droplets. These energy stores are reactivated by lipolysis, the enzymatic hydrolyzation of free fatty acids from triglyceride (13). Adipose triglyceride lipase (ATGL) is the first and rate-limiting enzyme of intracellular lipolysis. ATGL-derived fatty acids act as essential PPARα agonists (16–18). PPARs are nuclear receptors involved in the modulation of several aspects of the cellular energy metabolism (19). Upon ligand binding, PPARα drives the transcription of essential genes for fatty acid oxidation, mitochondrial biosynthesis, and function (16, 19, 20).

ATGL’s physiological importance is reflected in human neutral lipid storage disease with myopathy (OMIM #610717) (21, 22) and the severe phenotypes of ATGL-deficient mouse models (16, 23–27). *Atgl*‑KO mice die of cardiac insufficiency at a young age. Molecular analysis of their heart tissues show (a) reduced mitochondrial numbers, (b) reduced mitochondrial respiration, and (c) excessive triglyceride accumulation caused by decreased fatty acid oxidation. Cardiac transgenic *Atgl* (cTg) overexpression in *Atgl*-KO/cTg mice (*Atgl*^–/–,Myh6Atgl+/–^) rescued the Atgl-KO phenotype. *Atgl*‑KO/cTg mice are healthy and have neither a cardiac phenotype nor a reduced lifespan (16, 28). We found, however, that they develop lung cancer at an older age (23). Although ATGL’s importance for cellular energy-metabolism is relatively well understood (13, 16, 28), its importance for the lung is still underinvestigated.

In summary, bronchiolar epithelial maintenance and repair are energy-intensive processes (29–31) that are of critical importance for lung health (1–5), and ATGL is crucial for cellular energy supply (13, 16, 28). Hence, we hypothesized that ATGL protects the lungs in an unknown manner. Therefore, we set out to specifically analyze the function of ATGL in the airways, using the *Atgl*‑KO/cTg mouse strain.

## Results

### ATGL deficiency causes bronchiolar triglyceride accumulation and increases airway resistance.

**Figure 1:**
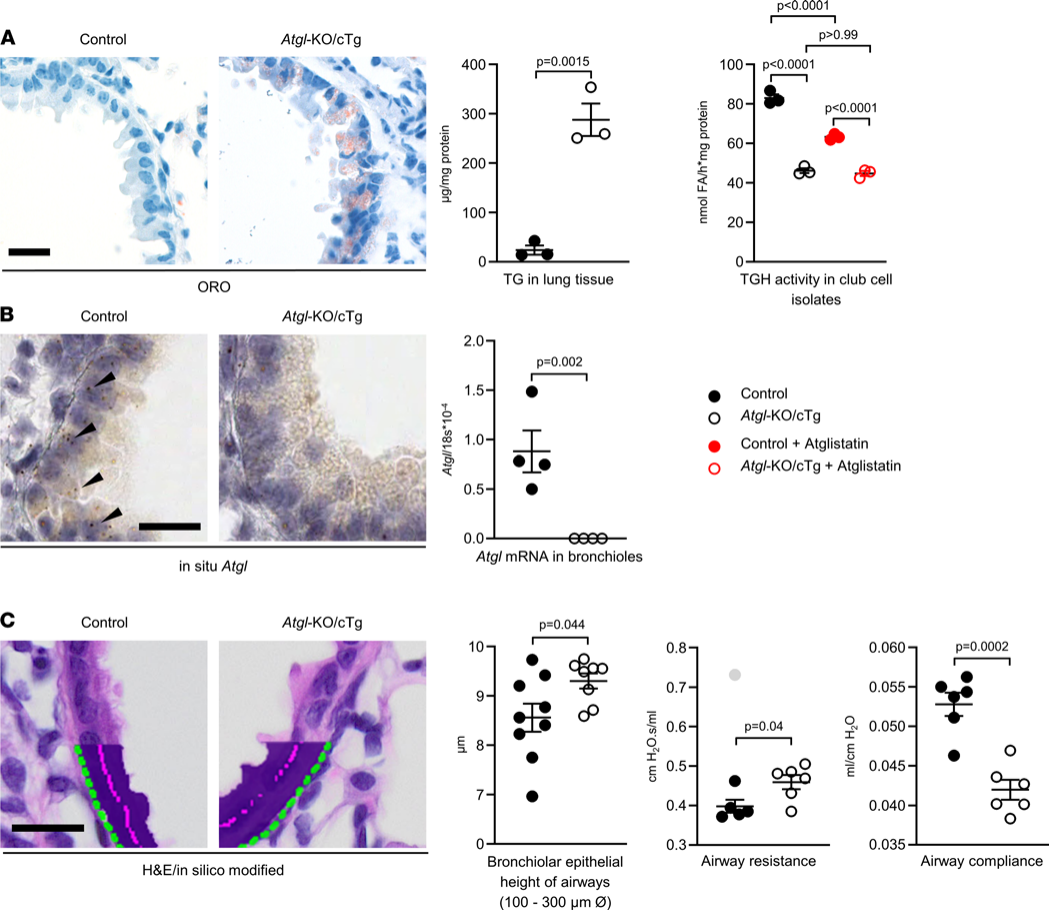
Airways of *Atgl*-KO/cTg mice show triglyceride accumulation and elevated breathing resistance. (**A**) Representative bronchioles in lung sections from control and *Atgl*‑KO/cTg mice stained with ORO. Scatter plots report data on triglyceride (TG) in lung tissues (*n* = 3 mice/group), and TGH activity in club cell isolates. The ATGL inhibitor Atglistatin was present if indicated. *n* = 3 experiments per group. We used 1-way ANOVA with Bonferroni’s test for multiple comparisons. (**B**) In situ *Atgl*: Images of lung sections incubated with *Atgl* mRNA–specific probes. Arrowheads depict representative hybridization signals. The scatter plot shows *Atgl* mRNA expression in LCM bronchioles determined by qPCR and normalized to 18S rRNA. *n* = 4 mice/group. (**C**) Representative H&E sections. Images were computationally modified to illustrate the bronchiolar height measurement process. Scatter plots report data on bronchiolar epithelial height of airways (*n* = 9 control mice and *n* = 8 Atgl-KO/cTg mice). Airway function parameters, resistance and compliance, were measured using a computer-controlled piston ventilator (*n* = 6 mice/group). Animals were aged 6 to 9 months. Error bars depict SEM. Statistical analysis was performed with Student’s 2-tailed *t* test. The outlier (gray) was detected using Grubb’s test (α = 0.05).Scale bars: 20 μm. Detailed information on animals is provided in Supplemental Table 1.

Because ATGL is needed for triglyceride lipolysis (25), we first assessed the neutral lipid content of the bronchiolar epithelium of mice lacking ATGL. Lung sections of *Atgl*‑KO/cTg mice stained with the neutral lipid dye Oil-Red-O (ORO), showed strong signals in the bronchiolar epithelia, whereas those of isogenic controls (*Atgl*^+/+,Myh6Atgl+/–^) were negative (Figure 1A). Lipidomics revealed triglyceride accumulation in the lungs of *Atgl*‑KO/cTg mice (Table 1), and biochemical measurements showed that the triglyceride concentration in their lungs was 12‑fold higher (288 μg/mL) than in those of control mice (24 μg/mL) (Figure 1A). One reason for this may be that isolated club cells from *Atgl*‑KO/cTg mice only had approximately half the triglyceride hydrolase (TGH) activity (46 nmol fatty acids/h × mg protein) as compared to controls (83 nmol fatty acids/h × mg protein) (Figure 1A). Pretreatment with the specific ATGL inhibitor Atglistatin significantly inhibited TGH activity in control club cell isolates (83 vs. 63 nmol fatty acids/h × mg protein), whereas no Atglistatin effect was seen in those from *Atgl*‑KO/cTg mice (46 vs. 45 nmol fatty acids/h × mg protein) (Figure 1A) (32–34). As expected, *Atgl* mRNA was undetectable by in situ hybridization in bronchioles of *Atgl*‑KO/cTg mice. In bronchioles of isogenic control mice, however, in situ *Atgl* mRNA staining was even stronger than in the surrounding alveolar cells (Figure 1B). For quantification of *Atgl* mRNA, we extracted bronchioles by laser capture microdissection (LCM) (Supplemental Figure 1; supplemental material available online with this article), isolated RNA, and performed real-time PCR (qPCR). Compared with 18S rRNA, we found 0.9 × 10^–4^ *Atgl* mRNA molecules in control bronchi but none in *Atgl*‑KO/cTg bronchi (Figure 1B). Lipid accumulation was associated with a significantly thicker bronchiolar epithelium in *Atgl*‑KO/cTg mice (9.3 μm) than in control mice (8.6 μm) (Figure 1C). This morphological difference was reflected by functional measurements. We detected enhanced airway resistance (0.461 vs. 0.398 cm H_2_O.s/mL), and reduced airway compliance (0.042 vs. 0.053 mL/cm H_2_O), in *Atgl*‑KO/cTg lungs compared with control lungs (Figure 1C).

### ATGL is essential for normal club cell metabolism.

**Figure 2:**
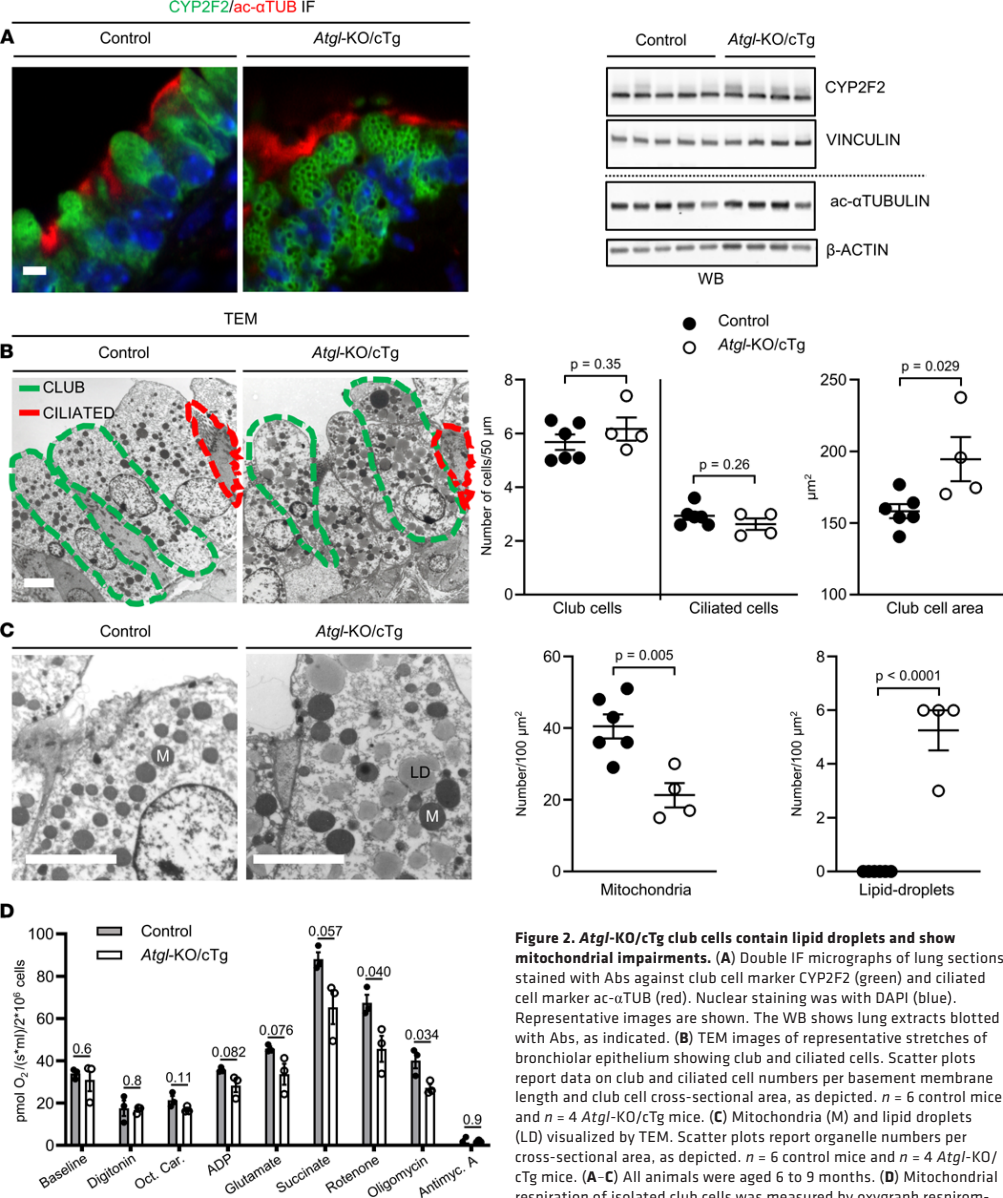
*Atgl*‑KO/cTg club cells contain lipid droplets and show mitochondrial impairments. (**A**) Double IF micrographs of lung sections stained with Abs against club cell marker CYP2F2 (green) and ciliated cell marker ac‑αTUB (red). Nuclear staining was with DAPI (blue). Representative images are shown. The WB shows lung extracts blotted with Abs, as indicated. (**B**) TEM images of representative stretches of bronchiolar epithelium showing club and ciliated cells. Scatter plots report data on club and ciliated cell numbers per basement membrane length and club cell cross-sectional area, as depicted. *n* = 6 control mice and *n* = 4 *Atgl*-KO/cTg mice. (**C**) Mitochondria (M) and lipid droplets (LD) visualized by TEM. Scatter plots report organelle numbers per cross-sectional area, as depicted. *n* = 6 control mice and *n* = 4 *Atgl*-KO/cTg mice. (**A**–**C**) All animals were aged 6 to 9 months. (**D**) Mitochondrial respiration of isolated club cells was measured by oxygraph respirometry. Specific substrates for respective mitochondrial complexes were added sequentially, as depicted. *n* = 3 experiments/group. Mice were aged 3 to 4 months. Error bars depict SEM. Statistical analysis was performed using Student’s 2-tailed *t* test. Scale bars: 5 μm. Detailed information on animals is provided in Supplemental Table 1. ADP, adenosine diphosphate; Antimyc., antimycin; Oct. Car., octenoylcarnitine.

We next assessed whether loss of ATGL causes changes in the cellular composition of the bronchiolar epithelium. To assess the relative club cell and ciliated cell fractions, we performed CYP2F2 and ac-αTUB immunofluorescence analyses of lung tissue sections (Figure 2A). Additionally, we performed CYP2F2 and ac-αTUB Western immunoblotting (WB) to quantify these club cell and ciliated cell markers, respectively (Figure 2A). Both immunofluorescence and WB revealed no differences in the expression of CYP2F2 and ac-αTUB between lungs from both genotypes. Finally, we used transmission electron microscopy (TEM) to count both cell types along the bronchiolar basement membranes (Figure 2B). This confirmed a physiological 2:1 distribution between club cells and ciliated cells, regardless of genotype (7). However, in accordance with increased epithelial thickness (Figure 1C), the average cross-sectional area of club cells in *Atgl*‑KO/cTg mouse lungs (195 μm^2^) was larger than that of cells from control lungs (158 μm^2^) (Figure 2B).

Healthy club cells usually contain a high number of mitochondria scattered throughout their cytoplasm (35–38), and cells lacking ATGL often develop big cytoplasmic lipid droplets (25, 27). Indeed, high-power TEM analysis enabled us to identify mitochondria and lipid droplets in bronchiolar club cells (Supplemental Figure 2A). On average, 40.5 mitochondria were visible per 100 μm^2^ in control club cells, whereas those of *Atgl*‑KO/cTg, typically contained about half as many (21.3 mitochondria/100 μm^2^) (Figure 2C). Lipid droplets, on the other hand, were abundant in club cells of *Atgl*‑KO/cTg mice (5.3 lipid droplets/100 μm^2^), whereas none were observed in club cells of control mice (Figure 2C). This finding is consistent with lipid staining and quantification (Figure 1A and Table 1) and with the pitted, porous structure of the green CYP2F2^+^ IF staining, which is indicative of intracellular spherical structures in *Atgl*‑KO/cTg club cells (Figure 2A). One reason for reduced mitochondrial numbers could be their selective degradation by mitophagy (autophagy). Nevertheless, ultrastructures indicative of mitophagy and autophagy (autophagosomes) were detected at statistically indistinguishable numbers in both genotypes, using TEM (Supplemental Figure 2B) (39, 40). Similarly, lipid droplet production in nonadipose cells may be linked to ER stress. However, no indication of enhanced splicing of the ER stress marker *Xbp1* could be detected in LCM-isolated bronchiolar epithelial cells of either genotype (Supplemental Figure 2C) (41).

We next assessed mitochondrial respiration in isolated club cells (16, 33, 42). Measurements under unstimulated conditions (baseline) showed no differences in oxygen uptake between club cell isolates from both genotypes. When we stimulated mitochondrial complex II (succinate) and inhibited complex I (rotenone), however, a significantly higher oxygen‑uptake rate was observed in control cells [67 pmol/(s × mL)/2 × 10^6^ cells] compared with *Atgl*‑KO/cTg ([46 pmol/(s × mL)/2 × 10^6^ cells]. A significant difference still persisted [40 vs. 27 pmol/(s × mL)/2 × 10^6^ cells] upon ATP‑synthase/complex-V inhibition (oligomycin) (Figure 2D).

### The airway epithelium in mice lacking ATGL has reduced regeneration potential.

**Figure 3:**
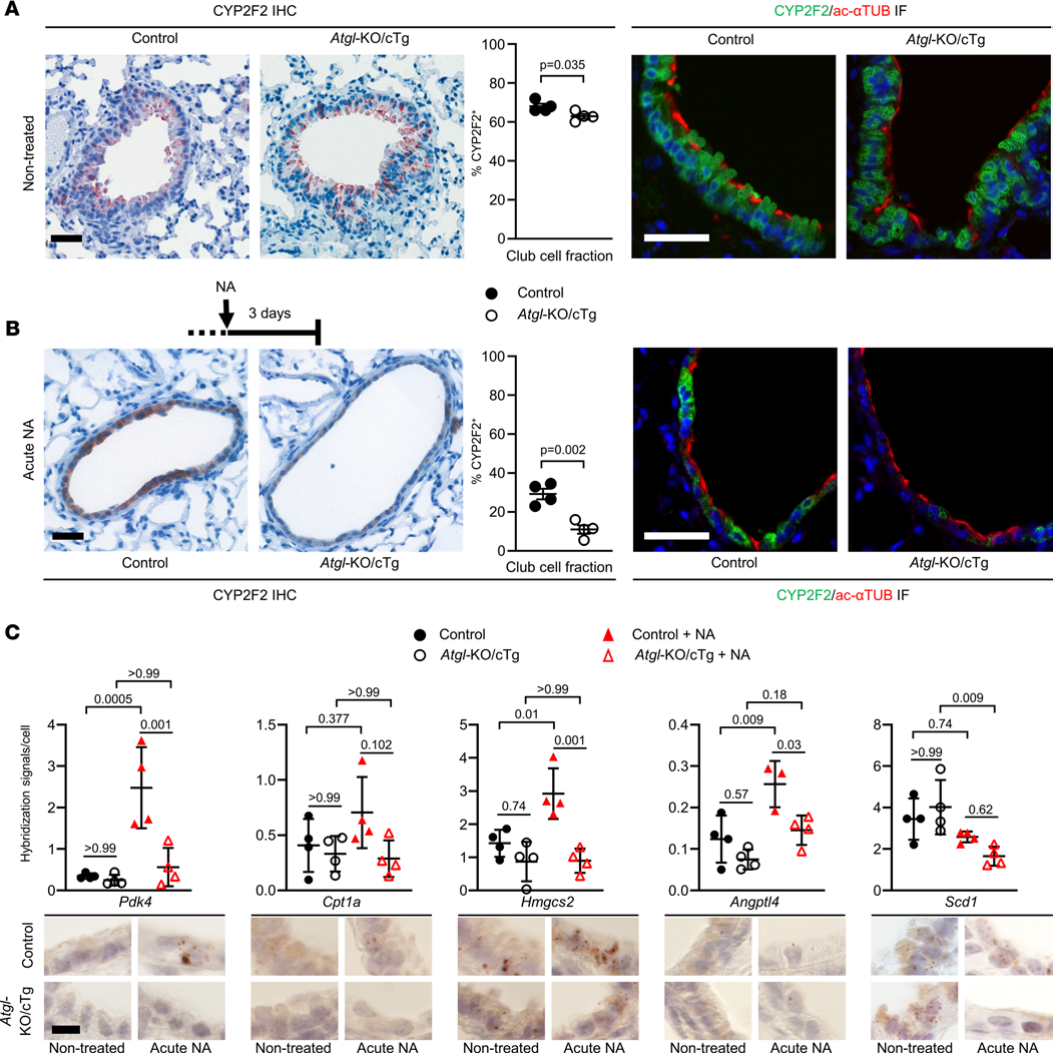
*Atgl*-KO/cTg mice have impaired bronchiolar club cell regeneration. (**A**) CYP2F2 IHC: Lung sections of nontreated control mice and *Atgl*‑KO/cTg mice, stained with club cell marker Ab CYP2F2 (brown), nuclear staining, and hematoxylin (blue). The scatter plot shows the relative fraction of CYP2F2^+^ cells. *n* = 4 mice/group. Double IF micrographs show lung sections stained with CYP2F2 (green) and ac‑αTUB (red) Abs. Nuclear staining was with DAPI (blue). (**B**) Acute NA–treated animals were sacrificed at day 3 after treatment and analyzed as described for **A**. *n* = 4 mice/group. (**C**) Lung sections from nontreated or Acute NA–treated mice, incubated with in situ hybridization probes against PPARα target mRNAs, as depicted. Scatter plots report data from quantification of the hybridization signals in bronchioles. *n* = 4 mice/group, except the control plus NA of *Angptl4* group, *n* = 3 mice. Representative images are shown. Animals were aged 6 to 9 months. Scale bar: 40 μm (**A** and **B**); 10 μm (**C**). Error bars depict SEM. Statistical analysis was conducted with Student’s 2-tailed *t* test (**A** and **B**) or 1-way ANOVA with Bonferroni’s test for multiple comparisons (**C**). Detailed information on animals is provided in Supplemental Table 1.

In healthy animals, club cells metabolically detoxify xenobiotics and regenerate the bronchiolar epithelium following chemical injury (8–10). Due to the lipid accumulation, reduced mitochondrial numbers, and decreased cellular respiration, we hypothesized that ATGL-deficient club cells might be unable to fulfill their physiological roles in the bronchiolar epithelium. To test this, mice were exposed to a sublethal dose of NA, which denudes the airways by depleting club cells, and the regenerative response was quantified by CYP2F2 IHC (7–12). Although untreated *Atgl*-KO/cTg mice had only a slightly lower fraction of CYP2F2^+^ cells along the bronchiolar basement membrane (63%) than isogenic controls (68%) (Figure 3A), a much stronger phenotype was apparent 3 days after NA treatment. Here, the relative content of CYP2F2^+^ bronchiolar cells was 29% in control mice but only 11% in *Atgl*‑KO/cTg mice (Figure 3B). As reported before, ciliated cells filled the spaces between the regrowing CYP2F2^+^ club cells (Figure 3B) (10, 11), indicating a bronchiolar epithelial regeneration defect in *Atgl*‑KO/cTg mice. The relative number of actively proliferating 5-ethynyl-2′-deoxyuridine–positive (EdU^+^) bronchiolar epithelial cells, however, was higher in the *Atgl*‑KO/cTg group than in the control group, indicating ongoing epithelial repair (Supplemental Figure 3A).

To understand potential differences in their modes of NA degradation, we incubated club cell isolates with NA and traced its metabolic products, 1- and 2-naphthol (43). Relative naphthol production was statistically indistinguishable between club cells with or without ATGL (Supplemental Figure 3B).

To determine whether the absence of ATGL-derived fatty acids led to reduced PPARα signaling (16, 18) in bronchiolar epithelia of *Atgl*‑KO/cTg mice, we measured the expression of 5 PPARα target genes using in situ mRNA hybridization. At steady state (i.e., nontreated animals), we did not detect any significant differences between the 2 genotypes. Three days after NA treatment (acute NA), however, 3 of the 5 probes (*Pdk4*, *Hmgcs2*, and *Angptl4*) showed significantly stronger hybridization signals in bronchiolar epithelia of the control group, as compared with the *Atgl*‑KO/cTg group (Figure 3C). The other 2 probes (*Cpt1a* and *Scd1*) showed a similar trend (Figure 3C). Moreover, in control mice, *Pdk4, Hmgcs2*, *and Angptl4* mRNAs were significantly upregulated 3 days after NA treatment, whereas no such induction occurred in the *Atgl*‑KO/cTg group (Figure 3C). These data indicate a potential role of PPARα signaling in bronchiolar regeneration, which is awaiting further investigation.

To examine the long-term effects of repeated club cell ablation, we treated animals once per week with NA, for 4 weeks. Thereafter, the epithelium was left to regenerate for 2.5 months (chronic NA). Chronic NA-treated control mice had a high amount of CYP2F2^+^ cells in their bronchi (77%), which was indicative of successful airway epithelial regeneration. Bronchi of chronic NA-treated *Atgl*‑KO/cTg mice, however, had less-well-regenerated airways, and only 43% of all their bronchiolar cells were CYP2F2^+^. Moreover, we regularly noted areas that were completely devoid of CYP2F2^+^ cells and covered by long stretches of apically ac-αTUB^+^ ciliated cells (Supplemental Figure 3C).

### Pharmacological PPARα activation corrects mitochondrial dysfunction in club cells of ATGL-deficient mice.

**Figure 4:**
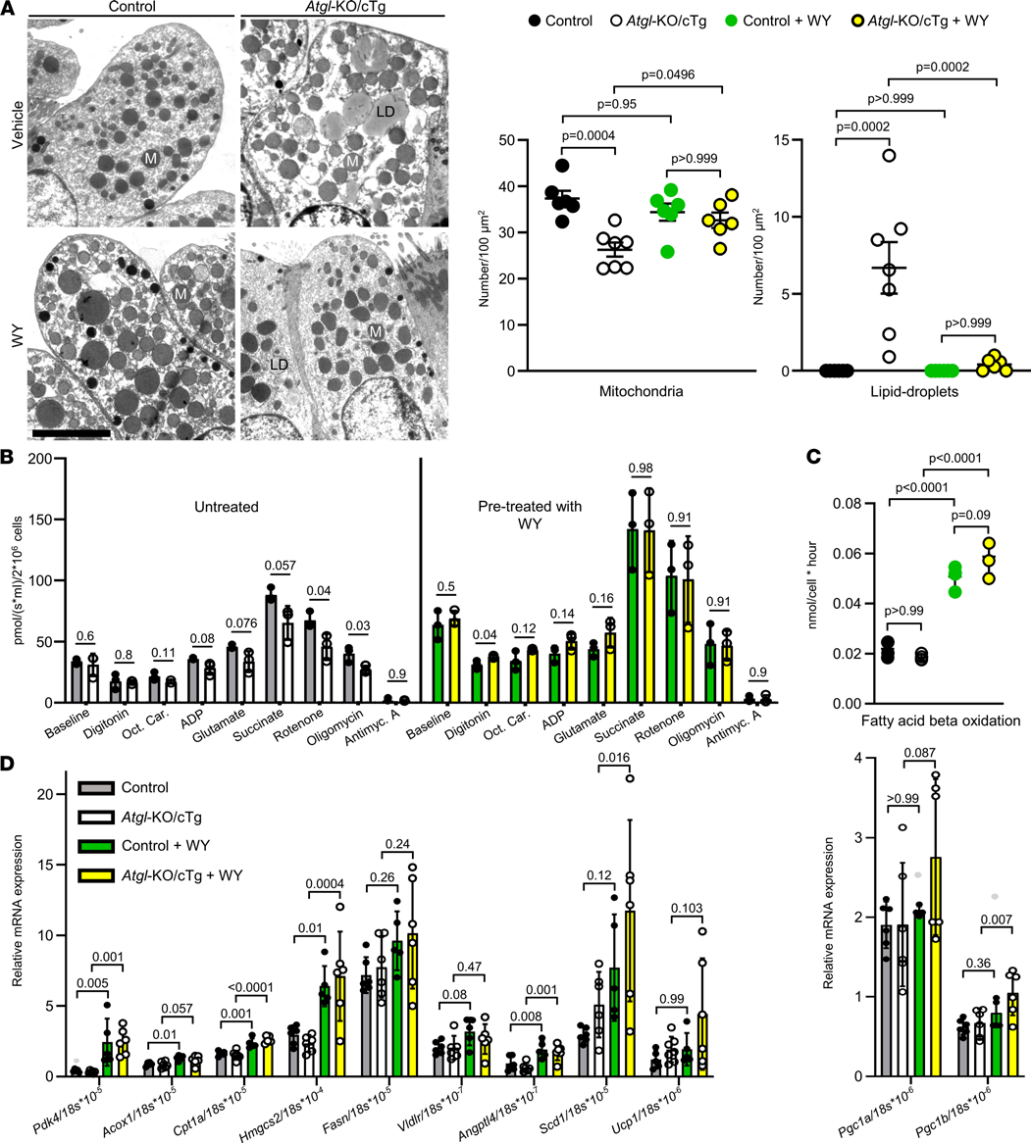
PPARα agonist rescues the mitochondrial club cell phenotype of *Atgl*-KO/cTg. Mice were pretreated with WY for 2 weeks (**A** and **D**) or 5 days (**B** and **C**), if indicated. (**A**) TEM images of club cells. The scatter plots report mitochondrial (M) and lipid droplet (LD) counts. *n* = 6 mice/group, except *Atgl*-KO/cTg, *n* = 7 mice. (**B**) Oxygraph respirometry data of isolated club cells are reported. Mitochondrial complex-substrates were added, as depicted. *n* = 3 experiments/group. Untreated groups are also shown in Figure 2D. (**C**) Fatty acid β-oxidation of isolated club cells. *n* = 3 experiments/group. (**D**) PPARα target gene expression in LCM-isolated bronchioles, measured by qPCR, and normalized to 18S rRNA. *n* = 6 mice/group, except control + WY (*n* = 5 mice) and *Ucp1* of *Atgl*‑KO/cTg (*n* = 7 mice). Mice were aged 9 to 11 (**A** and **D**) or 3 to 5 (**B** and **C**) months. Error bars depict SEM. Statistical analyses were performed by ordinary 1-way ANOVA with Bonferroni’s test for multiple comparisons, except in **B**, where Student’s 2-tailed *t* test was used. The outlier (gray) was detected using Grubb’s test (α = 0.05). Detailed information on animals is provided in Supplemental Table 1. ADP, adenosine diphosphate; Antimyc., antimycin; Oct. Car., octenoylcarnitine.

Club cells of *Atgl*‑KO/cTg mice had fewer mitochondria and reduced mitochondrial function (Figure 2). Whole-body *Atgl*-KO mice have a similar mitochondrial defect in cardiac myocytes that is caused by reduced PPARα signaling (16). Therefore, we hypothesized that the mitochondrial phenotype in bronchiolar club cells of *Atgl*‑KO/cTg mice may be rescued by forced PPARα activation. We gavaged *Atgl*‑KO/cTg and control mice for 14 days with the potent and specific PPARα agonist WY14643 (WY) or vehicle. Subsequently, we analyzed the bronchiolar epithelial ultrastructure by TEM (Figure 4A). On average, club cells of vehicle-treated control mice had 37 mitochondria/100 μm^2^ viewing field, as compared with 26 mitochondria/100 μm^2^ viewing field in cells of *Atgl*‑KO/cTg mice. Two weeks of gavage with WY, however, significantly elevated the numbers of mitochondria to, on average, 33 mitochondria/100 μm^2^ viewing field in cells of *Atgl*‑KO/cTg mice, whereas they remained unaltered (34 mitochondria/100 μm^2^ viewing field) in club cells of control animals (Figure 4A). The number of lipid droplets in *Atgl*-KO/cTg club cells was significantly reduced from 7 lipid droplets/100 μm^2^ viewing field to 0.4 lipid droplets/100 μm^2^ viewing field, by virtue of WY gavage (Figure 4A).

We next assessed the effect of the PPARα agonist on mitochondrial respiration. In club cells isolated from untreated *Atgl*‑KO/cTg animals, significantly reduced oxygen uptake under mitochondrial complex II stimulation (with succinate) and mitochondrial complex I inhibition (with rotenone), as well as under mitochondrial complex V inhibition (with oligomycin) occurred compared with cells from control mice (Figure 2D and Figure 4B). If mice were pretreated, however, with WY for only 5 days, we no longer could detect any statistically significant difference between the 2 genotypes (Figure 4B). Thus, pharmacological activation of PPARα signaling by WY rescued mitochondrial respiration in club cells lacking ATGL. Moreover, WY apparently led to a general increase in oxygen consumption in both genotypes (Figure 4B).

PPARα signaling reportedly activates lipid oxidation (16); hence, we incubated club cell isolates from untreated and mice pretreated for 5 days with WY with ^14^C-labeled palmitate and monitored the production rate of radiolabeled acid-soluble metabolites to measure fatty acid β-oxidation (44). Indeed, pretreatment with WY significantly increased the β-oxidation rate in both genotypes (from 0.022 to 0.051 nmol/cell × h in control cells, and from 0.019 to 0.059 nmol/cell × h in club cells lacking ATGL) (Figure 4C). To assess if WY activated PPARα target gene expression in the bronchiolar epithelium, we performed LCM of the bronchiolar compartment (Supplemental Figure 1). The majority of PPARα target genes analyzed were significantly upregulated by WY gavage in both genotypes (Figure 4D). Expression of PPAR co‑activators, *Pgc1a* and *Pgc1b*, remained largely unchanged except that *Pgc1b* mRNA increased upon WY treatment in *Atgl*‑KO/cTg bronchioles (Figure 4D).

### WY gavage restores the airway-regeneration capability in mice lacking ATGL.

**Figure 5:**
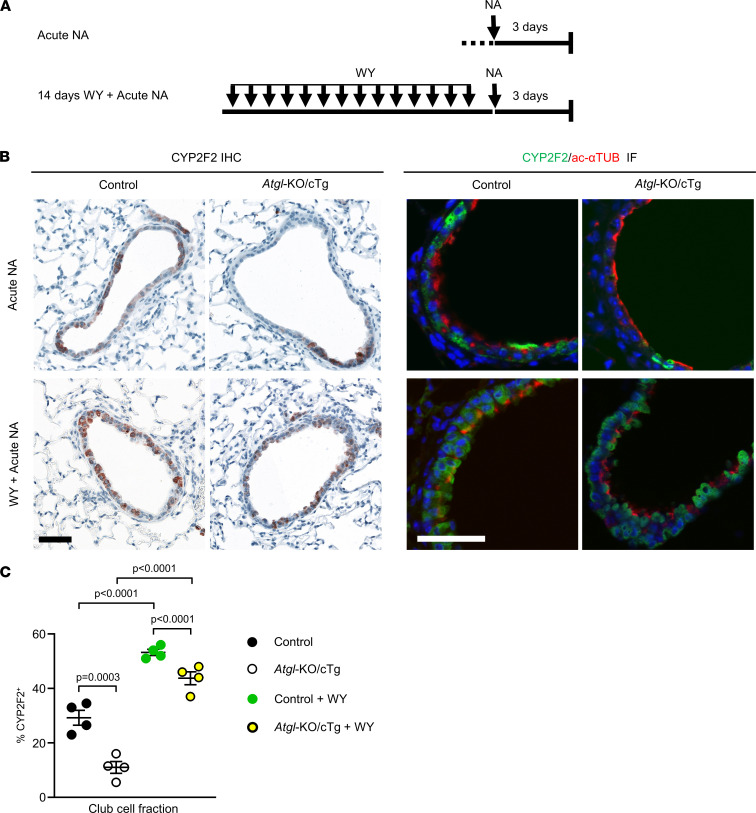
PPARα agonist treatment improves bronchiolar regeneration. (**A**) Animals were pretreated with WY for 14 days, if indicated. Next, all animals were treated with acute NA and sacrificed 3 days later. (**B**) CYP2F2 IHC: Lung sections from mice, as depicted, were stained with the club cell marker Ab CYP2F2 (brown); nuclear staining was with hematoxylin (blue). Double IF micrographs show lung sections stained with CYP2F2 (green) and ac‑αTUB (red) Abs. Nuclear staining was with DAPI (blue). (**C**) The relative fraction of CYP2F2^+^ bronchiolar cells. *n* = 4 mice/group. Mice were aged 6 to 9 months. Acute NA groups are the same as in Figure 3B. Error bars depict SEM. Statistical analyses were performed by ordinary 1-way ANOVA with Bonferroni’s test for multiple comparisons. Scale bar: 50 μm. Detailed information on animals is provided in Supplemental Table 1.

The PPARα target genes *Pdk4*, *Hmgcs2*, and *Angptl4* were significantly upregulated during bronchiolar regeneration in control mice but not in *Atgl*‑KO/cTg mice (Figure 3C). Additionally, pharmacological induction of PPARα signaling by WY gavage rescued the mitochondrial phenotypes of *Atgl*‑KO/cTg club cells (Figure 4). These findings led us to hypothesize that PPARα signaling is needed for optimal bronchiolar injury repair. To test this, we pretreated control and *Atgl*-KO/cTg mice with WY for 2 weeks and then applied NA to induce acute injury (Figure 5A). Three days later, we assessed the fraction of CYP2F2-expressing bronchiolar cells. Control mice had 53% CYP2F2^+^ bronchiolar cells instead of 29% without WY. *Atgl*‑KO/cTg mice had 44% CYP2F2^+^ bronchiolar cells, instead of 11% without WY (Figure 5, B and C). Moreover, the histological appearance of regenerating bronchiolar epithelia in *Atgl*‑KO/cTg mice normalized. Fewer denuded basement membrane-stretches (covered by ciliated cells) were found when we pretreated animals with WY (Figure 5B).

## Discussion

Dysregulation of energy homeostasis is a key feature of many pathophysiological processes. It is also a central characteristic of metabolic syndrome (45). Several defining clinical features of metabolic syndrome, central obesity, type 2 diabetes, hypertension, and enhanced blood triglyceride levels, have been linked to reduced lung function in human epidemiological studies (46–51). Moreover, an increasing body of evidence shows that cellular energy metabolism has a crucial role in lung epithelial regeneration and pathogenesis of respiratory diseases. For example, glucose uptake and glycolysis are critical factors for the proliferative expansion of club cells during airway epithelial repair (52). Cell differentiation and repair go hand in hand and are highly energy-consuming processes (31). Hence, it is not astonishing that impaired mitochondrial function underlies several chronic lung diseases, including pulmonary arterial hypertension, asthma, and chronic obstructive pulmonary disease (30), and hampers epithelial repair (29). Nevertheless, little is known about the role of lipid metabolism for bronchiolar epithelial repair.

ATGL-mediated lipid catabolism is crucial for mitochondrial biogenesis and cellular energy conversion (13, 16). Moreover, we have reported that mice lacking ATGL developed bronchiolar epithelial neoplastic lesions (23). Based on these findings, we hypothesized that ATGL is essential for the physiological function of the airway epithelium.

In this study, we reveal that bronchiolar club cells of *Atgl*-KO/cTg mice (which lack ATGL everywhere except for the heart) display mitochondrial defects, accumulate ectopic lipid droplets, and exhibit impaired bronchiolar epithelial regenerative capacity. Together, these data suggest that ATGL is an important factor for the regeneration of the airway epithelium, due to its importance in the energy metabolism of club cells. This notion is reinforced by what we know about ATGL’s function in other cell types. Whole-body *Atgl*-KO mice die early because of mitochondrial dysfunction and excessive lipid deposition in cardiac myocytes, due to abrogated PPARα signaling (16, 25, 53). Humans with neutral lipid storage disease with myopathy have skeletal muscle myopathy and often lethal cardiomyopathy, due to absence or reduction of ATGL activity (21, 22).

Our histological, biochemical, and lipidomics analyses demonstrated triglyceride accumulation in the lungs of *Atgl*‑KO/cTg mice. Their bronchiolar epithelial club cells exhibited less lipolytic activity than those from control animals. Instead, cells from *Atgl*‑KO/cTg mice had ectopic lipid droplets, fewer mitochondria, and reduced mitochondrial respiration. Furthermore, the overall number of club cells was slightly reduced when ATGL was absent, which may indicate a fundamental regenerative defect in the small airways. Indeed, via functional in vivo assays, we detected severely impaired airway regeneration in *Atgl*‑KO/cTg mice following NA-induced club cell abrogation. In vitro experiments with freshly isolated club cells showed no detectable difference in the NA degradation products 1- and 2-naphthol between club cells isolated from *Atgl*‑KO/cTg and control mice. This finding suggests that the recorded regeneration defect was not due to a difference in NA degradation ability.

In summary, we found that decreased club cell energy metabolism in *Atgl*-KO/cTg mice is accountable for impaired airway regeneration after NA challenge and links mitochondrial dysfunction to this impaired bronchiolar epithelial repair capacity. These findings support and expand on earlier studies that showed regeneration of small bronchioles in mice is driven by club cells (7) and mitochondrial function is important for efficient lung epithelial repair (14).

ATGL-mediated PPARα activation drives mitochondrial biogenesis and transcriptionally triggers a metabolic switch toward lipid β-oxidation (16, 20). In uninjured bronchiolar epithelia from *Atgl*-KO/cTg and control mice, however, we found no differences in PPARα target gene expression. This either indicates (a) that it is not needed under steady-state conditions or (b) that the expression of PPARα target genes in other bronchiolar cells (7) masks the changes in unstimulated club cells. In contrast, 3 days after NA challenge, 3 of 5 PPARα targets were significantly more weakly expressed in bronchiolar epithelium of *Atgl*‑KO/cTg mice compared with control mice. For the other 2 mRNAs, a similar trend was evident. However, this analysis may have been biased to some degree because in *Atgl*‑KO/cTg mice, epithelium regenerated slower and those mice had fewer club cells within their bronchi than did control mice. Nevertheless, again the same 3 (of 5) mRNAs (*Pdk4*, *Hmgcs2*, and *Angptl4*) were significantly upregulated in control bronchi regenerating from injury, as compared with uninjured bronchi, and no such upregulation was seen in *Atgl*-KO/cTg mice. It is conceivable, therefore, that important PPARα targets are only induced in the bronchiolar epithelium when needed (i.e., during repair) (54, 55). Remarkably, we detected significantly more EdU incorporation in regenerating bronchi of *Atgl*‑KO/cTg mice than in those of controls. At first glance, this finding sounds counterintuitive; however, it likely indicates that regenerative club cell expansion was still ongoing in *Atgl*‑KO/cTg epithelium while this step of regeneration was probably already almost finished in control mice (11).

Taken together, our data suggest that ATGL-mediated PPARα–driven target gene expression is essential for bronchiolar regeneration. This hypothesis is supported by the finding that treatment with a PPARα agonist (WY) fully restored mitochondrial numbers and corrected the ectopic lipid droplet accumulation in club cells lacking ATGL. WY treatment also boosted mitochondrial respiration and fatty acid β-oxidation in club cells. At the same time, WY treatment enhanced the expression of common PPARα target genes in bronchioles, indicating that the expected PPARα activation was also evident on a molecular level. Treatment with WY, prior to NA-induced airway denudation, fully rescued the bronchiolar regeneration defect of *Atgl*‑KO/cTg mice. In addition, in both genotypes, *Atgl*‑KO/cTg and control mice, WY pretreatment led to quicker regeneration than in untreated control mice. Prolonged consumption of diets rich in specific fatty acids reportedly stimulate PPARα signaling (56). Future studies thus may address the beneficial effects of differential feeding regimens on lung regeneration.

Altogether, our findings pointed to PPARα as the important molecular link between lack of ATGL activity, lipid droplet accumulation, and impaired club cell energy metabolism due to decreased mitochondrial function and impaired bronchiolar epithelial regeneration potential. We suggest that normally functioning lipolysis is of key importance for the bronchiolar epithelium. It provides energy-rich fatty acids, activates lipid β-oxidation, and mitochondrial biogenesis through PPARα signaling (16, 18). Thereby, ATGL-driven lipolysis creates essential requirements for normal bronchiolar epithelial regeneration.

Whether lack of ATGL confers a defect in all types of bronchiolar club cells, or rather in a specific subset of them, cannot be fully answered with our set of experiments. Variant club cells, for example, show low expression of CYP2F2 and a stem-cell-like phenotype. They are thought to survive NA exposure and to repopulate the bronchiolar epithelium after denudation by NA (57, 58). One observation that would argue for the exhaustion of variant club cells in *Atgl*‑KO/cTg mice is the patchy appearance of their bronchiolar epithelium after chronic NA treatment (11). Hence, tracing of pulmonary linage-specific phenotypes, due to *Atgl* absence, is an important future task.

ATGL-mediated triglyceride catabolism not only activates mitochondrial fatty acid catabolism through PPARα but can also activate the lipogenic nuclear-receptor PPARγ (28). Moreover, the PPARγ agonist rosiglitazone had a strong therapeutic effect on experimentally induced pulmonary fibrosis (59, 60) and inflammation (61). This, and the notion that lipogenically differentiated myofibroblasts counteract lung fibrosis (62), make it very attractive to investigate ATGL also in a broader pulmonary context.

In summary, our results show that lipid metabolism is essential for bronchiolar epithelial regeneration, with a strong involvement of PPARα signaling. Although lipogenesis and lipogenic differentiation of specific lung cell linages are already a focus of lung regenerative biology (59–63), not much is currently known about the importance of lipolysis for the lung. With our work, we highlight the significance of ATGL-mediated triglyceride catabolism for lung health and lung regeneration.

## Methods

### Animals.

*Atgl*-deficient animals with *Atgl* expression in the heart, *Atgl*^–/–,Myh6Atgl+/–^ (MGI:3629035 × MGI:5294356) were designated as *Atgl*-KO/cTg mice. Mice were provided in-house, and the mice were bred as previously described (16). All experiments were carried out with male and female mice on a fully backcrossed (≥10 times) C57BL/6JRj background. Our control groups consist of *Atgl*^+/+,Myh6Atgl+/–^ mice that are isogenic to *Atgl*-KO/cTg experimental mice. In some cases, to reduce breeding numbers, we used WT (*Atgl^+/+^*) mice as controls and *Atgl*-KO mice without cTg (*Atgl^–/–^*) that were otherwise isogenic to the *Atgl*-KO/cTg experimental mice; for in vitro experiments with isolated club cells, see Supplemental Table 1. Generally, all animals were kept on a regular light/dark cycle (14-h light/10-h dark) at 22 ± 1°C in a specific pathogen-free environment and were fed a standard laboratory chow diet ad libitum.

### Lung function measurements.

Mice were anesthetized deeply using 150 mg/kg ketamine and 20 mg/kg xylazine, followed by intubation and mechanical ventilation (150 breaths/minute, tidal volume of 10 mL/kg, and a positive end-expiratory pressure of 2 cm H_2_O). Airway resistance, compliance, and total lung capacity were measured using a FlexiVent apparatus (SciReq), as previously described (64).

### NA studies.

For acute NA(Sigma-Aldrich, 184500) treatment, NA was dissolved in corn oil and injected i.p. at a dose of 200 mg/kg and 130 mg/kg BW into male and female mice, respectively. On the second day after NA treatment, mice were injected i.p. with EdU (Thermo Fisher Scientific, E10187) dissolved in PBS at the dose of 50 mg/kg BW. Mice were sacrificed on day 3 after NA injection. Chronic NA treatment was performed when the mice were 6 to 7 months old. Mice received 1 i.p. NA injection per week for the first 4 weeks of the experiment (male mice: week 1: 200 mg/kg, weeks 2–4: 80 mg/kg; female mice: week 1: 130 mg/kg, weeks 2–4: 50 mg/kg), followed by 10 weeks of aging without treatment (7–12).

### Club cell isolation.

Club cells were isolated based on the protocol described by Oreffo et al. (33) with several modifications. Briefly, mice were anesthetized using 100 mg/kg ketamine and 20 mg/kg xylazine and sacrificed by rupturing the dorsal vein. Lungs were perfused venously with PBS, lavaged 3 times with solution I (133 mM NaCl, 5.2 mM KCl, 2.59 mM phosphate buffer at pH 7.4, 10.3 mM HEPES buffer at pH 7.4, 1 mg/mL glucose, 5 mM EDTA) and instilled with solution I containing 0.25% (wt/vol) trypsin. Next, lungs were perfused under constant flow (3 mL/minute) with solution I containing 0.25% (wt/vol) trypsin in a 37°C water bath for 15 minutes. Afterward, the main bronchi were removed, the rest of the lung was cut into approximately 1 mm^3^ pieces, and these were transferred into 10 mL of PBS/lung. Bronchial epithelial cells were released by shaking for 1 minute. Thereafter, 1 mL of FBS was added, and cells were filtered through a 100 μm and 40 μm cell strainer and centrifuged for 10 minutes at 190*g*. The pellet was resuspended in 600 μL of PBS and loaded onto a 1.04 g/mL to 1.09 g/mL iodixanol density step gradient. After centrifugation in a Beckman Coulter Optima L-90K ultracentrifuge (20 minutes at 1422*g* at 4°C), the club cell enriched fraction was recovered. Cells were either subjected to immune cytochemistry, RNA isolation, protein analysis by WB, TGH assay, β-oxidation assay, NA detoxification assay, or oxygen-consumption measurements.

### PPARα agonist gavage.

WY (Cayman Chemical Co., 70730) was dissolved in DMSO and mixed with olive oil as a vehicle. For mitochondrial- and lipid droplet–number rescue experiments (Figure 4), 177 mg WY/kg BW/day was gavaged. If mice lost more than 15% of their BW, gavaging was suspended for 1 day. Thereafter, the animals received 50%, and later 75%, of the normal dose until their BW again was within the 15% margin. Animals in the control group were given oil and DMSO vehicle only. If WY gavage was suspended due to BW loss, vehicle treatment was suspended similarly in respective control animals. Treatment was carried out for 14 days. For NA regeneration experiments, 43 mg/kg BW/day was used. Treatment was carried out for 14 days, and on the 15th day, mice were i.p. injected with NA dissolved in corn oil (male mice, 200 mg/kg; female mice, 130 mg/kg) and sacrificed after 3 days of treatment. For mitochondrial respiration rescue experiments and fatty acid β-oxidation experiments, mice were pretreated with 83 mg/kg BW/day WY for 4 days and 176 mg/kg BW/day on day 5, 4 hours before sacrifice.

### Oxygen consumption.

The oxygen-consumption rate of isolated club cells (pooled club cell isolates from 3–5 mice, yielding 2–4 × 10^6^ cells) was measured with the Oroboros O2k-FluoRespirometer instrument in a respiration buffer containing 125 mM sucrose, 20 mM K-Tes, 2 mM MgCl_2_, 1 mM EDTA, 4 mM KH_2_PO_4_, 3 mM malate, and 0.1% (wt/vol) BSA at pH 7.2. Cells were permeabilized using 7 μg of digitonin and the following substrates and inhibitors were added consecutively when the oxygen slope was stable: 0.2 mM octanoylcarnitine, 1 mM adenosine diphosphate, 10 mM glutamate, 10 mM succinate, 0.5 μM rotenone, 4 μg/mL oligomycin, and 2.5 μM antimycin. The oxygen consumption rate was normalized to the cell number, as depicted in each figure.

### LCM.

Mice were anesthetized using 100 mg/kg ketamine and 20 mg/kg xylazine and sacrificed by rupturing the dorsal vein. Lungs were perfused venously with 0.9% (wt/vol) NaCl, lavaged twice with 1 mL of 0.9% (wt/vol) NaCl, and subsequently inflated with 0.6 mL of 50% (vol/vol) Tissue Plus OCT compound in PBS, 10% (wt/vol) sucrose (containing 10 U/μL RNase inhibitor; New England BioLabs lnc., RNase inhibitor, murine) and cut into several pieces (approximately 10 mm^3^ each). Lung pieces were placed in cryomolds with OCT compound to form a capsule on dry ice. Capsules were immediately transferred to liquid nitrogen by wrapping them in aluminum foil and later stored at –80°C until use.

For tissue sectioning, frozen capsules with lung tissue were sectioned at a thickness of 20 μm. A minimum of 15 to 20 frozen sections were placed on Membrane Slides (Leica, No. 11505158, PEN-membrane 2.0 μm). Staining sections were stained in sterile-filtered hematoxylin stain for 20 seconds. Slides were rinsed in deionized water thereafter for 1 minute and dehydrated in an ascending ethanol gradient of 50%, 70%, and then 100% (wt/vol). Slides were air dried completely and used for LCM (65). LCM was performed on a Veritas Microdissection Instrument (Model 704, Arcturus Bioscience, lnc.; CapSure Macro LCM Caps, MDS Analytical Technologies), according to the manufacturer’s protocol. Isolated bronchioles on the membrane cap were immediately put in a 0.5 mL Eppendorf tube containing 200 μL of TRIzol and snap-frozen in liquid nitrogen by turning the tube upside down to make sure TRIzol was touching the membrane of the cap. Tubes were stored at –80°C until use. RNA was isolated according to the RNA Cleanup Kit (NEB, T2030L) protocol and also used for qPCR analysis.

### qPCR analysis.

RNA was isolated according to the RNA Cleanup Kit (NEB, T2030L) protocol, cDNA was prepared using the High-Capacity cDNA Reverse Transcription Kit (Applied Biosystems, 4368814), and qPCR was performed using the SYBR Green Luna Universal q-PCR Master Mix (NEB, M3003) on the QuantStudio 7 Flex Real-Time PCR System from Applied Biosystems (4485701). First, the efficiency of 18S primers and of each primer set for each gene of interest (GOI) were determined by computational standard curve analysis (QuantStudio 7 Flex Software). Relative efficiencies were calculated by dividing GOI by 18S RNA; as a base for dCT equations, the square of this value was used. For data analysis, we computed the square of the base minus dCT for each qPCR reaction. Primers were designed with the National Center for Biotechnology Information primer-designing tool and are listed in Supplemental Table 2.

### Tissue preparation and WB analysis.

Mice were anesthetized using 100 mg/kg ketamine and 20 mg/kg xylazine and sacrificed by rupturing the dorsal vein. Lungs were perfused venously with 0.9% (wt/vol) NaCl, lavaged twice with 1 mL of 0.9% (wt/vol) NaCl, excised, washed with 1× PBS, and subsequently snap-frozen in 2-methylbutane cooled in liquid nitrogen. Lung tissue pieces were lysed in SDS lysis buffer (100 mM Tris, pH 6.8, 3% SDS) using MagNA Lyser Green Beads (Roche MagNA Lyser, 25 seconds at 7000 rpm). Afterward, the tubes were centrifuged in a tabletop centrifuge (5 minutes at 16,200*g* at 4°C) and the supernatant transferred to a new Eppendorf tube. The lysates were sonicated (Hielscher Ultrasonics, 10 seconds at room temperature), heated (5 minutes at 70°C), and again centrifuged in a tabletop centrifuge (5 minutes at 16,200*g* at room temperature). The supernatant was then transferred to a new Eppendorf tube and protein concentration was determined using a Bio-Rad protein assay with BSA as a standard. Proteins of the lysates were separated using 4% to 20% SDS gels (Bio-Rad) and blotted onto a 0.45 μm nitrocellulose membrane (GE Healthcare). Membranes were stained with Ponceau S to verify efficient protein transfer and blocked with 5% skim milk. Proteins were detected using the primary Abs against CYP2F2 (Santa Cruz Biotechnology, sc-374540), ac-αTUB (Sigma, T7451), β‑actin (Sigma, A2228), vinculin (Sigma, hVIN-1), and their respective HRP-coupled secondary Abs (Dako, P0447 [Polyclonal Goat anti-Mouse Immunoglobulins/HRP]).

### Histological analyses.

Mice were anesthetized using 100 mg/kg ketamine and 20 mg/kg xylazine and sacrificed by rupturing the dorsal vein. Lungs were perfused venously with 0.9% (wt/vol) NaCl, and lavaged twice with 1 mL of 0.9% (wt/vol) NaCl. Next, all lobes needed for histological analyses were inflated with 4% (wt/vol) neutral buffered formalin and incubated for 24 hours. Afterward, lungs were embedded in paraffin (Tissue Tek Tec, Sakura), sectioned (4 μm), and stained with H&E according to standard histopathological techniques (66). IHC was performed using primary Abs against mouse CYP2F2 (Santa Cruz Biotechnology, sc-374540) and results were visualized using the Dual Link System-HRP (DAKO K4061) and the AEC Substrate Chromogen (DAKO K3464). IHCs were evaluated by counting the positive cells per bronchiole or per defined length of bronchiolar epithelium. At least 10 bronchioles of similar sizes per animal were examined. We also performed IF on deparaffinized slides of lung sections, using the Opal 4-Color IHC Kit (Akoya, NEL810001KT), according to the manufacturer’s protocol. The following Abs were used: CYP2F2 (Santa Cruz Biotechnology, SC374540) and ac-αTUB (Sigma, T7451). The following Ab dilutions were used: CYP2F2, 1:1000; and ac-αTUB, 1:2000.

To detect neutral lipids, lung tissue was resected, washed in 1× PBS, and fixed in 4% (wt/vol) paraformaldehyde for 4 hours. Lungs were transferred to 10% (wt/vol) sucrose for 16 hours and subsequently snap-frozen with 2-methylbutane in liquid nitrogen. Lungs were sectioned (4 μm) using Tissue Tek OCT compound and sections then were permeabilized using 0.1% (vol/vol) Triton X-100. Next, ORO staining (Sigma-Aldrich, O0625) was performed using a standard protocol (23). EdU incorporation was also detected on cryogenic lung sections, using the Click‑iT EdU Assay-Kit (Thermo-Fisher, C 10644) according to the manufacturer’s protocol.

### In situ hybridization.

In situ hybridization was performed using the Advanced Cell Diagnostics RNAscope 2.5 HD Detection Kit according to the manufacturer’s instructions and using the following Advanced Cell Diagnostics RNAscope probes: Mm-*Pnpla2* (catalog 469441) targeting region 1025–2115; Mm-*Pdk4* (catalog 437161) targeting region 834–1765; Mm-*Scd1* (catalog 461641) targeting region 2–2069; Mm-*Angptl4* (catalog 474611) targeting region 390–1432; Mm-*Cpt1a* (catalog 443071) targeting region 1234–2348; and Mm-*Hmgcs2* (catalog 437141) targeting region 2–861. Briefly, FFPE tissue sections were deparaffinized and after pretreatment, RNA-specific probes were hybridized to the target RNA. After signal amplification steps, visualization was performed using HRP-labeled probes and a chromogenic substrate. The PPIB gene and the bacterial gene dapB were used as positive and negative controls, respectively.

### Electron microscopy.

For electron microscopy, lung tissue was fixed in 2.5% (wt/vol) glutaraldehyde in 0.1 M cacodylate buffer at pH 7.3 for 3 to 4 hours. After dehydration, the tissue was embedded in resin (AGAR-100, Agar Scientific). Ultrathin lung sections (90 nm) were contrasted with uranyl acetate/lead citrate and studied with a Zeiss EM 900 electron microscope. Mitochondrial cristae were identified on micrographs made with a ThermoFisher (formerly, FEI) Tecnai G2 20 electron microscope, operated at 120 kV, with an Ametek (formerly, Gatan) Ultrascan 1000 camera.

### Triglyceride quantification.

Resected lung tissue (5–30 mg) was lysed in 1× PBS with MagNA Lyser Green Beads (Roche MagNA Lyser, 25 seconds at 7000 rpm). Afterward, the tubes were centrifuged in a tabletop centrifuge (1 minute at 16,200*g* at 4°C) and the supernatant transferred to a new microcentrifuge tube. The lysates were sonicated (Hielscher Ultrasonics, 3 times for 10 seconds at 4°C) and centrifuged again (1 minute at 16,200*g* at 4°C). A small amount of the supernatant was used to determine the protein concentration using a Bio-Rad protein assay, and the rest was transferred to a glass tube and used for lipid extraction according to Knittelfelder et al. (67). The lipid fraction was dissolved in 1% (vol/vol) Triton X-100 and TAG was measured using the Triglycerides Standard FS Kit (DiaSys) according to the manufacturer’s instructions. Triglyceride values were normalized to protein content.

### TGH assay.

Pooled club cell isolates of 4 mice were used for TGH assay. Club cells were washed twice in 1× PBS and suspended in 250 mL of assay buffer containing protease inhibitors on ice. After centrifugation (100*g* for 5 minutes at 4°C), the pelleted cells were disrupted by sonication (15% output, 10 seconds twice on ice). Cell lysate was centrifuged (1000*g* for 10 minutes at 4°C) and the supernatant was transferred to a new tube. The cell lysate was centrifuged (10,000*g* for 10 minutes at 4°C) and the fat-free infranatant taken using a hot needle. Club cell lysate lacking ATGL contained more fat on the top than did the control cell lysate. Protein was determined using Bradford reagent and BSA as standard (10 mL of sample plus 150 mL of reagent). Cell protein (35 μg) was subjected to TGH activity assay in the presence and absence of 20 μM Atglistatin (provided by Rolf Breinbauer, Graz University of Technology) using a radiolabeled 0.3 mM triolein substrate emulsified in a 3:1 ratio of phosphatidylcholine to phosphatidylinositol. After incubation for 1 hour at 37°C, the reaction was stopped and the fatty acids extracted using chloroform, methanol, and heptane, K_2_CO_3_. Radiolabeled fatty acids in the aqueous phase were determined by liquid scintillation counting.

### Fatty acid β-oxidation assay.

Pooled club cell isolates from 4 to 5 mice, pretreated for 5 days with PPARα agonist WY or left untreated, were used for each β-oxidation assay. Club cells were counted and centrifuged at 350*g* for 5 minutes at room temperature. The cell pellet was resuspended in 200 mL of STE buffer (250 mM sucrose, 10 mM Tris at pH 8, 1 mM EDTA plus 3.5 μg/mL digitonin plus the protease inhibitors pepstatin, leupeptin, and antipain, with or without WY [16 μg/mL] present). Fatty acid oxidation was measured using 1-^14^C palmitate, and acid-soluble metabolites and captured CO_2_ measurements were recorded, as mentioned elsewhere (44).

### NA detoxification assay.

Pooled club cell isolates of 4 mice were used for the NA detoxification assay. Freshly isolated club cells were washed in PBS. Club cells were then incubated at 37°C overnight in a shaker incubator with gentle agitation in 150 μL of PBS containing a saturated amount (1 mM) of NA (dissolved in DMSO) and NADPH (1 mM). Control tubes were incubated with cells without NA or in NA without cells. After the overnight incubation, tubes were ice cooled and 150 μL of methanol was added and mixed thoroughly. The mix was then centrifuged at 4°C for 15 minutes at 16 100*g* in an Eppendorf Centrifuge 5415R. Supernatant (200 μL) was transferred into glass vials with inserts and screw caps with Teflon septa and analyzed with a Shimadzu Nexera Ultra-High-Performance Liquid Chromatography (HPLC) system consisting of a DGU-20A5 Prominence degasser, LC-30AD pumps, SIL-30AC Nexera autosampler, CTO-20AC Prominence column oven, SPD-M20A Prominence photodiode array detector, LCMS-2020 single-quadrupole mass spectrometer equipped with an ESI source and a CBM-20A communications bus module, using an EC 150/3 Nucleodur C18 Gravity 3.0 μm (N0060018, batch 37408033; Macherey-Nagel) HPLC column. The analysis conditions were as follows: eluent A: water-containing 0.01 percentage by vol trifluoroacetic acid; eluent B: MeCN; 1.00 mL/min flow rate; at 40°C. A stepwise gradient was used as follows: from 0 to 0.7 minutes: A/B = 53:47; from 4.3 to 4.7 minutes: A/B = 95:5; from 4.71 to 6.5 minutes: A/B = 53:47. The injection volume was 10 μL, and UV detection was measured at 228 nm.

### Lipidomics analysis with mass spectrometry.

Total lipids of lung explants (5–30 mg) were extracted twice according to Folch et al. (68) using chloroform/methanol/water (2/1/0.6, vol/vol/vol) containing 500 nM butylated hydroxytoluene, 1% (vol/vol) acetic acid, and 4 nmol of internal standard mix (C17-lysophosphatidylcholine, C17-phosphatidylcholine, C17-triacylglycerol) per sample. Extraction was performed under constant shaking for 60 minutes at room temperature. The organic phase was collected after centrifugation (1000*g* for 15 minutes at room temperature). Combined organic phases of the double extraction were dried under a stream of nitrogen and dissolved in 200 μL of chloroform/methanol/2-propanol (2/1/12, vol/vol/vol) for UPLC-qTOF analysis. Chromatographic separation was performed using an AQUITY-UPLC system (Waters Corp.), equipped with an HSS T3 column (2.1 × 100 mm, 1.8 μm) as previously described (67). A SYNAPTG1 qTOF HD mass spectrometer (Waters Corporation) equipped with an ESI source was used for detection. Data acquisition was done with MassLynx, version 4.1, software (Waters Corp.). Lipid classes were analyzed with Lipid Data Analyzer, version 1.6.2, software (69). Extraction efficacy and lipid recovery were normalized using internal standards. Lipid profiling was performed in-house.

### Graphics.

The graphical abstract was drawn using artistic depictions from Servier Medical Art by Servier and licensed under a Creative Commons Attribution 3.0 unported license. Chemical structures were drawn using PerkinElmer ChemDraw, Professional Version 19.1.0.8.

### Statistics.

Each biological replicate was defined as a biological unit (e.g., a cell, a lung, a mouse). Biological replicate values were computed as the arithmetic mean value of technical replicate values. All data represent mean values of the biological replicates ± SEM. The number of biological replicates for each experiment is reported in the figure legends. Statistical analysis between 2 groups of biological replicates were performed using Student’s 2-tailed *t* test, using GraphPad Prism, version 8.2.0. For multiple comparisons of biological replicates, we used ordinary 1-way ANOVA with Bonferroni’s test for multiple comparisons, again using GraphPad Prism, version 8.2.0. In Figures 2D and 4B, reporting data from the oxygen consumption analysis, Student’s 2-tailed *t* test was used because each pair of bars represents an independent treatment and not just a consecutive time point. *P* < 0.05 was considered statistically significant. Outlier analysis was performed using Grubb’s test in GraphPad (https://www.graphpad.com/quickcalcs/grubbs1/), with α = 0.05.

### Study approval.

All animal studies were approved by and performed according to the guidelines of the Ethics Committee of the University of Graz and Medical University of Graz, the Austrian Federal Ministry for Science and Research, and are in accordance with the council of Europe Convention (ETS 123). Approved animal applications include BMWFW-66.007/0030-WF/V/3b/2015, BMWFW-66.007/0017-WF/V/3b/2016, BMBWF-66.007/0023-V/3b/2018, BMWFW-66.007/0016-WF/V/3b/2017, BMBWF-66.007/0005-V/3b/2018, BMBWF-66.007/0014-V/3b/2018, BMWFW-66.007/0035-WF/V/3b/2017, BMBWF-66.007/0007-V/3b/2018, and BMBWF-66.007/0016-V/3b/2018.

## Author contributions

MMK, ISB, and PWV designed, planned, and performed experiments; computed results; and wrote the manuscript. BIW, SS, and TOE planned and performed experiments and computed results. EW, AL, MS, MW, and IK planned and performed experiments. GL identified subcellular organelles using TEM. LMM, GK, RZ, and GH designed and planned experiments and wrote the manuscript. MMK and ISB share the first-author position because ISB performed experiments and data evaluations underlying Figures 1–3 and Supplemental Figure 3, and MMK performed experiments underlying Figures 1, 3, 4, and 5, and all Supplemental Figures, and data evaluations for the whole manuscript. Both authors were critically involved in manuscript preparation. The authorship order between them was assigned because MMK performed most of the experiments during the revision period.

## Supplementary Material

Supplemental data

Supplemental table 1

Supplemental table 2

## Figures and Tables

**Table 1:**
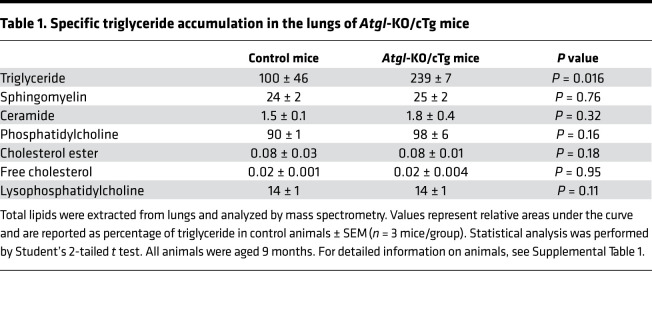
Specific triglyceride accumulation in the lungs of *Atgl*-KO/cTg mice

## Version 1 03/29/2022

In-Press Preview

## Version 2 05/09/2022

Electronic publication
