# Supplementary material for: Adipose triglyceride lipase–mediated lipid catabolism is essential for bronchiolar regeneration
Source: JCI Insight. 2022 May 9;7(9):e149438. doi: 10.1172/jci.insight.149438 (PMC9090255; doi:10.1172/jci.insight.149438)
Supplement: Supplemental data [file jciinsight-7-149438-s245.pdf]

Supplemental Figure 1. Laser capture microdissection (LCM).

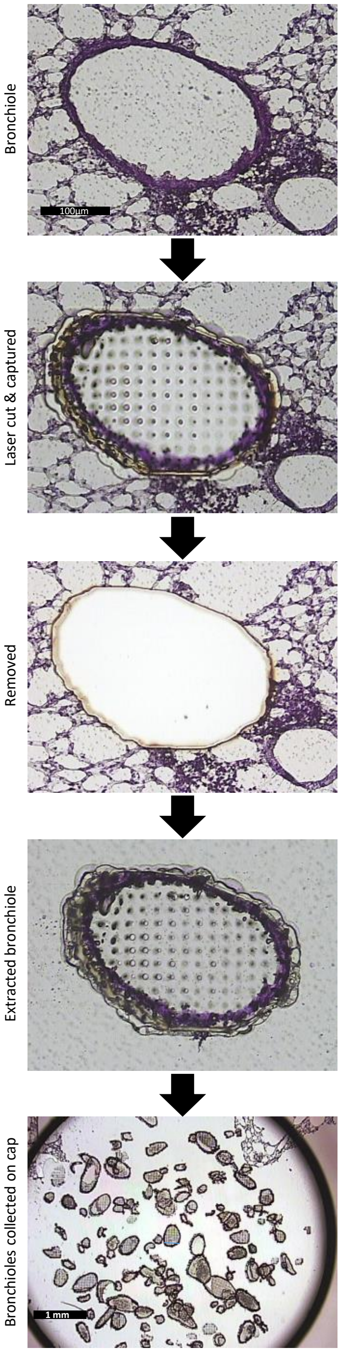

Bronchiole: Cryo-cut and Hämalaun stained lung section is placed on a membrane covered glass slide.

Laser cut & captured: a highly focused „cutting laser“ is used to cut along the circumference of the bronchiole. Next, a low power, „capture laser“, automatically tags the bronchiole to a membrane cap.

Removed: lung section devoid of bronchiole after LCM.

Extracted bronchiole: LCM captured bronchiole sticking to the membrane cap.

Bronchioles collected on cap: Cap with numerous bronchioles ready for RNA extraction.

For method validation we performed qPCR analyses that revealed a >25-fold enhancement of *Cyp2f2* mRNA when comparing LCM bronchioles to LCM alveolar tissue from a C57BL/6J WT mouse (data not shown). All scale bars represent 100 µm, except for the last image (Bronchioles collected on cap) where it represents 1 mm.

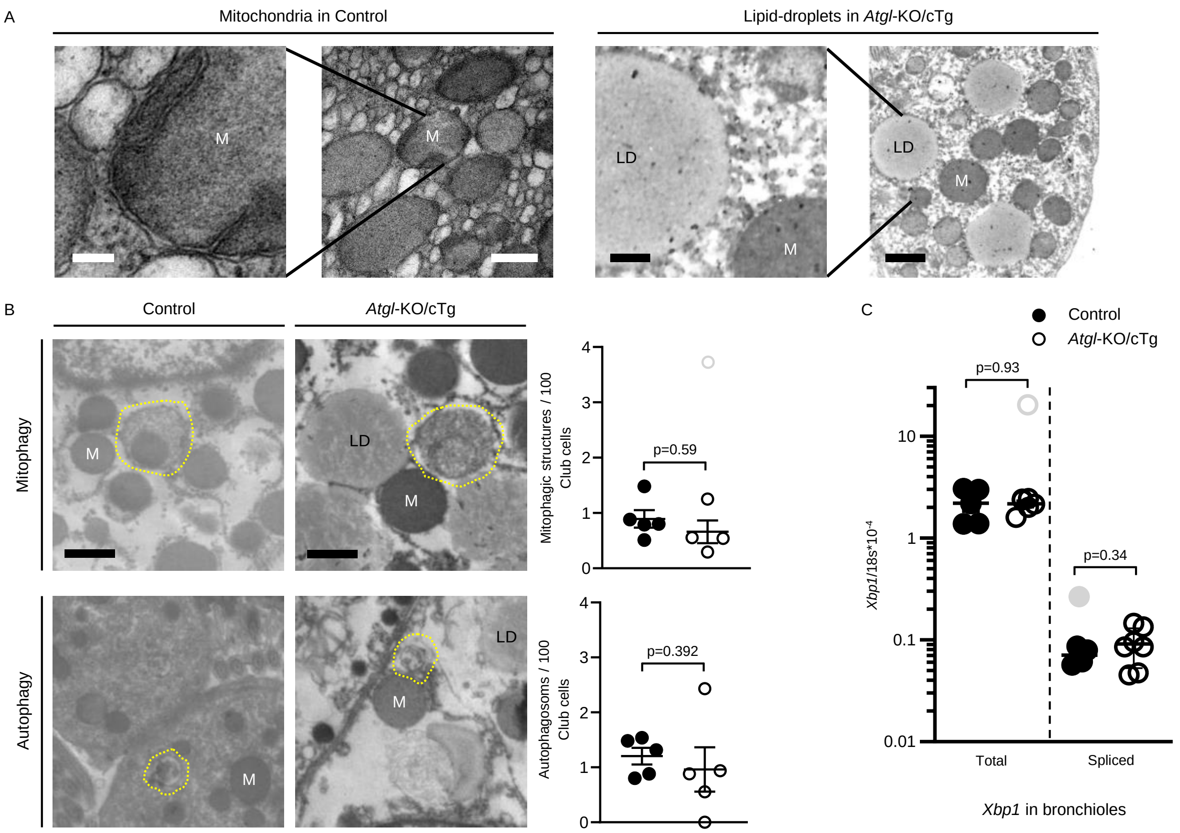

<u>Supplemental Figure 2. Autophagy/mitophagy and ER stress in club cells.</u>

(A) High magnification power Transmission electron microscopy (TEM) images were taken to confirm the identities of M (mitochondria) and LD (lipid-droplets). **(B)** Mitophagic and autophagic structures in club cells of control and *Atgl*-KO/cTg animals were visualized by TEM. Scatter plots: Numbers of mitophagic structures and autophagosomes per 100 club cell cross sections were statistically analysed. n=5/group. **(C)** Splice status of the ER stress marker mRNA *Xbp1* in bronchioles, isolated by LCM, was measured by qPCR. Total- and spliced- *Xbp1* mRNA is plotted normalized to *18s* rRNA. n=5 controls, and n=7 *Atgl*-KO/cTg. All animals aged 6-9 months. Error bars depict SEM. Statistical analysis was performed by Student's two-tailed t-test. Outlier (grey) detected using Grubb's test with alpha=0.05. Each Scale bar=1 µm, except (A). Here they depict, from left to right, 0.1, 0.5, 0.3, and 1 µm, respectively. Detailed information on animals (Supplemental Table 1).

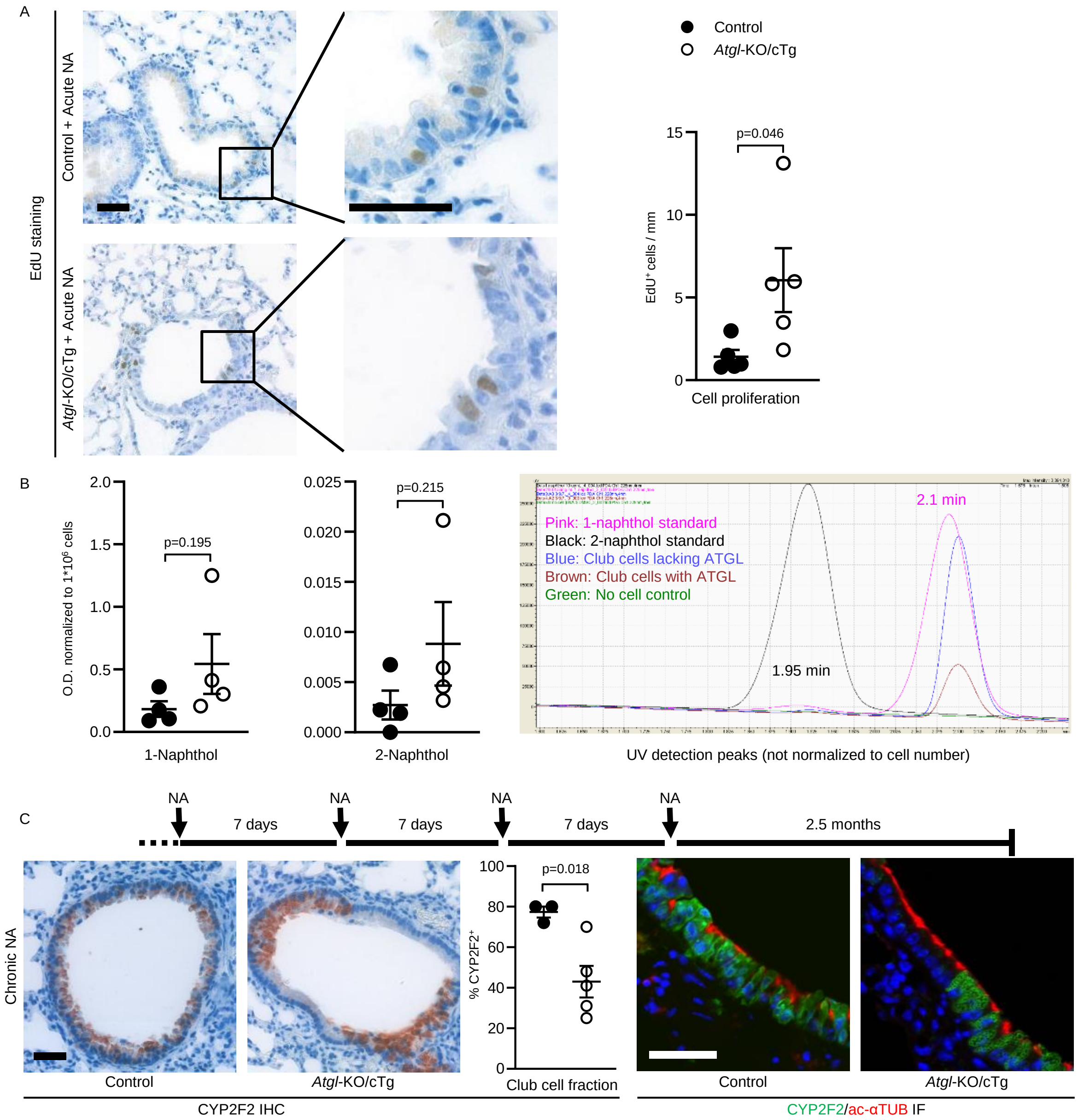

Supplemental Figure 3. Features of NA treated club cells

(A) Mice were injected with EdU (5-ethynyl-2'-deoxyuridine) 2 days post NA (Naphthalene) treatment and sacrificed 1 day later. Lung sections stained using Click-iT EdU Assay-Kit. Scatter plot: relative EdU^+ cell number in bronchioles. n=5/group. **(B)** Club cells isolated from mice, and in vitro incubated with NA over-night. NA degradation products, 1- and 2-naphthol measured using UV-spectroscopy (scatter plots). n=4/group. Detection-peaks and typical retention times see, example-image. **(C)** Mice treated as indicated in timeline, before sacrifice. CYP2F2 IHC: Lung sections stained with CYP2F2 antibody. Nuclear staining haematoxylin (blue). Scatter plot: Relative CYP2F2+ cell fractions in bronchioles. n=3 controls, and n=5 *Atgl*-KO/cTg. Double IF: Lung sections stained with antibodies against CYP2F2 (green) and ac αTUB (red). Nuclear staining DAPI (blue). Representative images shown. All animals 6-9 months old, except under (B) age=2 months. Error bars depict SEM. Statistical analysis by Student's two-tailed t-test. Each Scale bar=50 µm. Detailed information on animals (Supplemental Table 1).
